# Supplementary material for: Spatiotemporal dynamics of SETD5-containing NCoR–HDAC3 complex determines enhancer activation for adipogenesis
Source: Nat Commun. 2021 Dec 2;12:7045. doi: 10.1038/s41467-021-27321-5 (PMC8639990; doi:10.1038/s41467-021-27321-5)
Supplement: Supplementary file 1 — Supplementary information [file 41467_2021_27321_MOESM1_ESM.pdf]

Supplementary Information

Spatiotemporal dynamics of SETD5-containing NCoR-HDAC3 complex determines enhancer activation for adipogenesis

Yoshihiro Matsumura[1], Ryo Ito[2], Ayumu Yajima[1,3], Rei Yamaguchi[2], Toshiya Tanaka[4], Takeshi Kawamura[5], Kenta Magoori[1], Yohei Abe[1], Aoi Uchida[1], Takeshi Yoneshiro[1], Hiroyuki Hirakawa,[1,6] Ji Zhang[1,2], Makoto Arai[1,2], Chaoran Yang[2], Ge Yang[2], Hiroki Takahashi[2], Hitomi Fujihashi[1], Ryo Nakaki[7,8], Shogo Yamamoto[7], Satoshi Ota[7], Shuichi Tsutsumi[7], Shin-ichi Inoue[2], Hiroshi Kimura[9], Youichiro Wada[5], Tatsuhiko Kodama[4], Takeshi Inagaki[1,10], Timothy F. Osborne[11], Hiroyuki Aburatani[7], Koichi Node[3] & Juro Sakai[1,2]

[1]Division of Metabolic Medicine, Research Center for Advanced Science and Technology, The University of Tokyo, Tokyo 153-8904, Japan
[2]Division of Molecular Physiology and Metabolism, Tohoku University Graduate School of Medicine, Sendai 980-8574, Japan
[3]Department of Cardiovascular Medicine, Saga University, Saga 849-8501, Japan
[4]Department of Nuclear Receptor Medicine, Laboratories for Systems Biology and Medicine, Research Center for Advanced Science and Technology, The University of Tokyo, Tokyo 153-8904, Japan
[5]Isotope Science Center, The University of Tokyo, Tokyo 113-0032, Japan
[6]Department of Physiology and Cell Biology, Tokyo Medical and Dental University (TMDU), Graduate School, Tokyo 113-8510, Japan
[7]Genome Science Division, Research Center for Advanced Science and Technology, The University of Tokyo, Tokyo 153-8904, Japan
[8]Rhelixa Inc., Tokyo 101-0061, Japan
[9]Cell Biology Center, Institute of Innovative Research, Tokyo Institute of Technology, Yokohama 226-8503, Japan
[10]Laboratory of Epigenetics and Metabolism, Institute for Molecular and Cellular Regulation, Gunma University, Gunma 371-8512, Japan
[11]Institute for Fundamental Biomedical Research, Johns Hopkins All Children's Hospital, and Medicine in the Division of Endocrinology, Diabetes and Metabolism of the Johns Hopkins University School of Medicine, 600 Fifth Street S. St. Petersburg, FL 33701, USA.

These authors contributed equally: Yoshihiro Matsumura, Ryo Ito, Ayumu Yajima.

Correspondence and requests for materials should be addressed to Y.M. (email: matsumura-y@lsbm.org) or J.S. (email: jmsakai@med.tohoku.ac.jp)

Supplementary Tables

Supplementary Table 1. List of reagent and resource.

REAGENT or RESOURCE	SOURCE	IDENTIFIER
Antibodies		
Anti-H3K27ac mouse mAb	[1]	9E2H19
Anti-mouse SETD5 mouse mAb	This paper	IgG-F2104
Anti-mouse SETD5 mouse mAb	This paper	IgG-Z5721-234
Anti-TBP mouse mAb	Novus Biologicals	Cat#NB500-700, clone 1TBP18
Anti-V5 mouse mAb	Thermo Scientific	Cat#R960-25
Anti-Histone H3 rabbit pAb	Abcam	Cat#ab1791
Anti-HDAC1 mouse mAb	Santa Cruz Biotechnology	Cat#sc-8410, clone H-11
Anti-HDAC3 rabbit pAb	Abcam	Cat#ab7030
Anti-NCoR2 rabbit pAb	Abcam	Cat#ab5802
Anti-C/EBPβ rabbit pAb	Santa Cruz Biotechnology	Cat#sc-150x
Anti-C/EBPβ mouse mAb	Santa Cruz Biotechnology	Cat#sc-7962, clone H-7
Anti-CBP rabbit mAb	Cell Signaling Technology	Cat#7425, clone D9B6
Anti-H3K4me1 rabbit pAb	Abcam	Cat#ab8895
Anti-H3K4me3 rabbit pAb	Merck Millipore	Cat#07-473
Anti-Multi ubiquitin mouse mAb	Medical and Biological Laboratories	Cat#D058-3, clone FK2
Anti-ANAPC2 rabbit mAb	Cell Signaling Technology	Cat#12301
Anti-ANPC11 rabbit mAb	Cell Signaling Technology	Cat#14090, clone D1EQ7
Anti-CDC20 rabbit mAb	Cell Signaling Technology	Cat#14866, clone D6C2Q
Anti-Actin β mouse mAb	Sigma-Aldrich	Cat#A5441, clone AC15
Anti-Perilipin-1 rabbit mAb	Cell Signaling Technology	Cat#9349, clone D1D8
Anti-FLAG mouse mAb	Sigma-Aldrich	Cat#F3165, clone M2
Anti-mouse IgG-HRP	Sigma-Aldrich	Cat#A4416
Anti-rabbit IgG-HRP	Sigma-Aldrich	Cat#A0545
Bacterial plasmid and Virus Vector		
pMXs-IRES-Puro	Cosmo Bio	RTV-014
pMXs-IRES-Zeo	[2]	-

pMXs-Puro	Cosmo Bio	RTV-012
Chemicals, Peptides, and Recombinant Proteins		
DMEM High Glucose	Sigma-Aldrich	Cat#D6429
Fetal Bovine Serum	Thermo Fisher Scientific	Cat#10270
Penicillin-Streptomycin Mixed Solution	Nacalai Tesque	Cat#09367-34
3-Isobutyl-1-methylxanthine	Sigma-Aldrich	Cat#I5879
Dexamethasone	Sigma-Aldrich	Cat#D4902
Insulin	Sigma-Aldrich	Cat#I5523
Puromycin	Sigma-Aldrich	Cat#P8833
Zeocin	InvivoGen	Cat#ant-zn-1p
Oil Red O	Nacalai Tesque	Cat#25633-92
HydroMatrix Peptide Cell Culture Scaffold	Sigma-Aldrich	Cat#A6982
Super Signal West Dura Extended Duration Substrate	Thermo Fisher Scientific	Cat#34075
Lipofectamine RNAiMAX Transfection Reagent	Thermo Fisher Scientific	Cat#13778150
Stealth RNAi siRNA Negative Control Med GC Duplex #2	Thermo Fisher Scientific	Cat#12935-112
KOD Plus Neo DNA polymerase	Toyobo	Cat#KOD-401
ISOGEN Reagent	Nippon Gene	Cat#315-02504
Formaldehyde Solution	FUJIFILM Wako	Cat#064-00406
Sequencing Grade Modified Trypsin	Promega	Cat#V5111
Ethylene glycol bis(succinimidylsuccinate)	Thermo Fisher Scientific	Cat#21565
Dynabeads Protein G for Immunoprecipitation	Thermo Fisher Scientific	Cat#10004D
Protein G Sepharose 4 Fast Flow	GE Healthcare	Cat#17061801

Critical Commercial Assays		
HDAC Fluorometric Activity Assay Kit	Cayman Chemical	Cat#10011563
QIAquick PCR Purification Kit	Qiagen	Cat#28106
Qubit Double-stranded DNA High Sensitivity Assay Kit	Thermo Fisher Scientific	Cat#32854
TruSeq ChIP Library Preparation Kit	Illumina	Cat#RS-122-2001 or RS-122-2002
KAPA Hyper Prep Kit	Kapa Biosystems	Cat#KK8502
Histofine MAX-PO (R)	Nichirei Bioscience Inc.	Cat#414341
Deposited Data		
RAW and analyzed ChIP-seq data	This paper	GEO: GSE183849
Gene expression microarray data	This paper	GEO: GSE183849
Experimental Model: Cell Line		
3T3-L1	ATCC	ATCC CL-173
Plat-E	Cosmo Bio	RV-101
HEK293	ATCC	ATCC CRL-1573
Sf9	ATCC	ATCC CRL-1711
Software and Algorithms		
MACS1.4.2	[3]	http://liulab.dfci.harvard.edu/MACS/
MACS2.2.7.1	[3]	https://github.com/taoliu/MACS
SICER1.1	[4 5]	https://home.gwu.edu/~wpeng/Software.htm
SICER2-1.0.2	[4 5]	https://zanglab.github.io/SICER2/
DAVID6.7	[6]	https://david-d.ncifcrf.gov/
HOMER4.10.4	[7]	http://homer.ucsd.edu/homer/
Bowtie2.3.4.3	[8]	http://bowtie-bio.sourceforge.net/bowtie2/index.shtml
Trimmomatic0.39	[9]	http://www.usadellab.org/cms/?page=trimmomatic

Samtools1.9	[10]	http://www.htslib.org/
Bedtools2.27.1	[11]	https://bedtools.readthedocs.io/en/latest/
DEseq2.1.30.1	[12]	https://bioconductor.org/packages/release/bioc/html/DESeq2.html
ImageJ1.53k	-	https://imagej.nih.gov/ij/

Supplementary Table 2. List of RT-qPCR primers.

Gene	Forward primer	Reverse primer
Setd5	5'-CGTCGCCCGTAGAGGAACGC-3'	5'-CCCCTGCTCATTCCCCTGCACT-3'
Cebpa	5'-AGATGAGGGAGTCAGGCCGT-3'	5'-CGGAAAGTCTCTCGGTCTCAA-3'
Pparg	5'-CAAGAATACCAAAGTGCGATCAA-3'	5'-GAGCTGGGTCTTTTCAGAATAATAAG-3'
Ncor2	5'-CAAAAGGTCCCCAGAACCCAG-3'	5'-TTCCCCGTCTCGATACAGCAG-3'
Cebpb	5'-CAAGAAGACGGTGGACAAGCTGAG-3'	5'-GACAGCTGCTCCACCTTCTTCTG-3'
Cebpd	5'-TCGACTTCAGCGCCTACATTGAC-3'	5'-CCGCTTTGTGGTTGCTGTTGA-3'
Anapc2	5’-CCCAGGCTGACCAGAAGGAA-3’	5’-CCGGAGCATGCTGTAGATACG-3’
Anapc11	5’-GCTCTGGGTAGCCAATGATGAG-3’	5’-GCAGTCATCACCAGGCACCTT-3’
Cdc20	5’-GTCACGGCTTTGCCCAGAAC-3’	5’-GACCCGTGCTGTGTGTCCTT-3’
Viral Cebpa	5’-GTGGACCATCCTCTAGACTGCCG-3’	5’-TCTGGAGGTGACTGCTCATCGGG-3’
Human PPARG	5’-CATGGCAATTGAATGTCGTGTC-3’	5’-TGATTGTTCTCCGGAAGAAACC-3’

Supplementary Table 3. List of ChIP-qPCR primers.

Gene	Forward primer	Reverse primer
Cebpa -0.8 kb	5'-GTGGTGGTGTCCCGAACACT-3'	5'-CAGGCCAGAGCGATAGGATT-3'
Cebpa + 1 kb	5'-AACAGCAACGAGTACCGGGT-3'	5'-CGTCTCCACGTTGCGTTGT-3'
Cebpa + 8 kb	5'-CAGCTGGACTTAGTTGCCAAGC-3'	5'-TCTGACGGGTCCTAACCTGATG-3'
Cebpa +19 kb	5'- GCCCCACCAGGGATCTAGA-3'	5'- GGAAATGAGTCAGACAGCCATAAA-3'
Cebpa +35 kb	5'-CCCCAATCTTCCCTCAAATGA-3'	5'-GGAGCCCGGAACCAGAA-3'
Cebpa +53 kb	5'-TACCGCCGTTTCCCAAAC-3'	5'-TACCGCCGTTTCCCAAAC-3'
Pparg +60 kb	5'-GTTAGCAGTTTGGCACAGCTAGG-3'	5'-ATCAGGAAAACTCTGGCTTCTTG-3'
Pparg +158 kb	5'-TCCACAGAACAGGGCGATG-3'	5'-AAAGAAACCCAGCCCAGGC-3'
Hoxc13	5'-TGTCCTCCAGGGCTAAGGA-3'	5'-AGGTAGCCGGGCATTGC-3'
Ppib	5'-CTCACCCCAACTAGTCTAATCC-3'	5'-GTGACACACAGTGACTAACTTCC-3'

Supplementary Figure 1

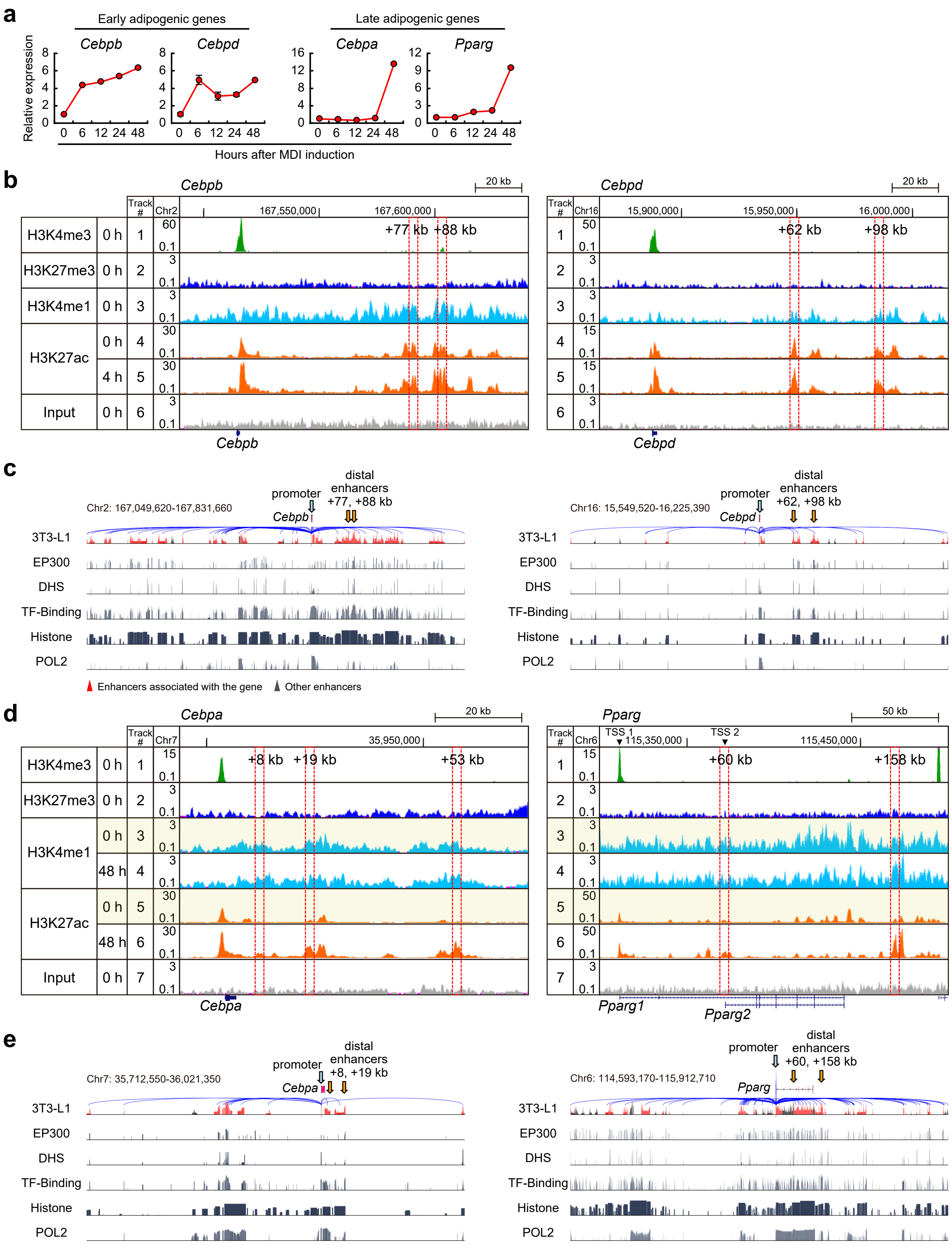

(continued on next page)

Supplementary Figure 1

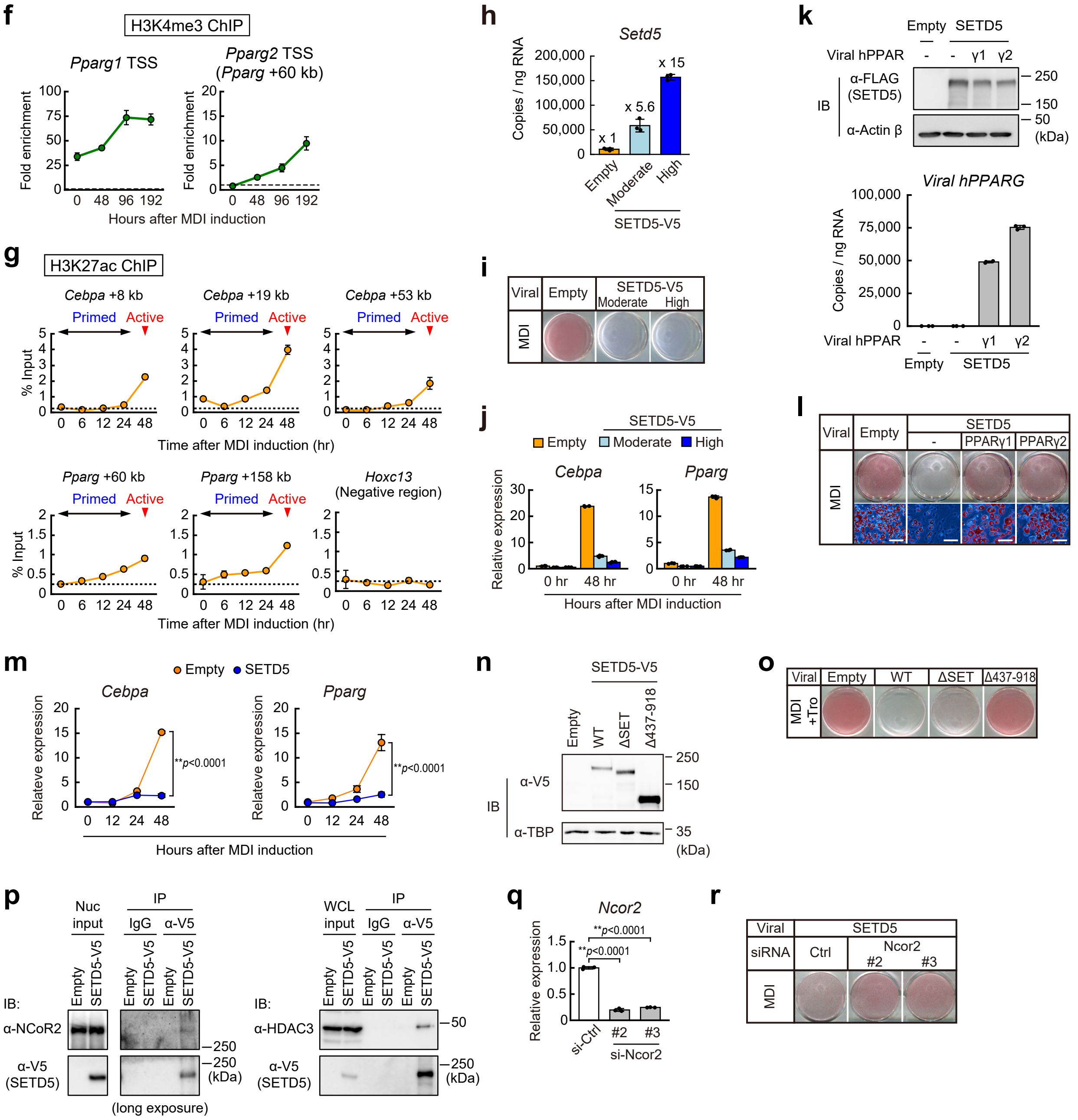

(continued from the previous page)

Supplementary Fig. 1 SETD5 associates with NCoR and HDAC to inhibit *Cebpa* and *Pparg* induction during adipogenesis. **a** Expression of mRNAs of adipogenic genes. Expression of mRNAs for early adipogenic genes (*Cebpb* and *Cebpd*) and late adipogenic genes (*Cebpa* and *Pparg*) were quantified by qPCR. **b, d** Genome browser representation for H3K4me3, H3K27me3, H3K4me1, and H3K27ac on *Cebpb* and *Cebpd* genes (**b**) and *Cebpa* and *Pparg* genes (**d**). 3T3-L1 preadipocytes were subjected to ChIP-seq at the indicated day of differentiation. ChIP-seq data for H3K4me3, H3K27me3, and input are from the previously published paper [13]. ChIP-seq data for H3K27ac in panel (**b**) were from the previously published paper [14] [15]. **c, e** Enhancer Atlas 2.0 [16] genome browser presentation of distal enhancers of *Cebpb* and *Cebpd* genes (**c**) and *Cebpa* and *Pparg* genes (**e**) in 3T3-L1 cells. EP300, E1A-associated protein p300; DHS, DNase I hypersensitive site; TF, transcription factor; POL2, RNA polymerase II. **f** ChIP-qPCR analysis of H3K4me3 on *Pparg* gene during adipogenesis. 3T3-L1 preadipocytes were subjected to ChIP-qPCR analysis using anti-H3K4me3 antibody. **g** ChIP-qPCR analysis of H3K27ac on *Cebpa* and *Pparg* genes during the early adipogenesis. 3T3-L1 preadipocytes were subjected to ChIP-qPCR analysis using anti-H3K27ac antibody. Data are mean ± SEM of three independent experiments. Primed enhancer (low H3K27ac) and active enhancer (high H3K27ac) are highlighted. **h** Retroviral expression of SETD5-V5 in 3T3-L1 preadipocytes. *Setd5* mRNA expression was quantified based on standard curves generated by using serial dilutions of plasmid DNA containing SETD5. **i** ORO staining of 3T3-L1 preadipocytes transduced with empty virus or SETD5-V5. Preadipocytes were induced with MDI mixture **j** Transcriptional changes of *Cebpa* and *Pparg* in SETD5-transduced 3T3-L1 preadipocytes during early adipogenesis. mRNA expression was quantified by qPCR. **k** Retroviral expression of FLAG-tagged SETD5 and human PPARγ1 or PPARγ2. Whole-cell lysate from 3T3-L1 preadipocytes transduced with the indicated retroviruses was subjected to immunoblot (IB) analysis (*upper*). Equal loading of the proteins was confirmed by blotting with anti-actin β antibody. mRNA expression of human *Pparg* was quantified by qPCR (*lower*). **l** ORO staining of 3T3-L1 preadipocytes transduced with empty virus or FLAG-tagged SETD5 or together with human PPARγ1 or PPARγ2. Preadipocytes were induced for differentiation with MDI mixture. Scale bar = 100 μm. **m** Expression of mRNA of *Cebpa* and *Pparg* during the early adipogenesis. 3T3-L1 preadipocytes transduced with empty or SETD5 retrovirus were induced for differentiation with

MDI. mRNA levels at the indicated times were quantified by qPCR. Data are mean ± SEM of three independent experiments. **n** Retroviral expression of SETD5 or mutants in 3T3-L1 preadipocytes. Nuclear proteins from 3T3-L1 preadipocytes transduced with empty virus, SETD5-V5, or mutants were subjected to immunoblot (IB) analysis. Equal loading of the proteins was confirmed by blotting with anti-TBP antibody. **o** ORO staining of 3T3-L1 preadipocytes transduced with empty virus, SETD5, or mutants. Preadipocytes were induced with MDI mixture troglitazone (Tro). **p** Co-immunoprecipitation of SETD5 with NCoR2 and HDAC3. Nuclear fraction (Nuc) or whole-cell lysate (WCL) from 3T3-L1 preadipocytes transduced with SETD5-V5 was subjected to immunoprecipitation using anti-V5 antibody followed by immunoblot (IB) analysis using anti-NCoR2, anti-HDAC3, or anti-V5 antibody. **q** siRNA-mediated knockdown of NCoR2. ON-TARGETplus siRNA targeting to *Ncor2* and control siRNA was transfected into SETD5-transduced 3T3-L1 preadipocytes, and *Ncor2* mRNA expression was quantified by qPCR. **r** ORO staining of NCoR2 knocked-down, SETD5-transduced 3T3-L1 preadipocytes. Preadipocytes were induced with MDI mixture. **a, j, l, n, p** Representative of three (**a**, **j**, **n**) or two (**l**, **p**) independent experiments. **a, f, h, j-k, q** Data are mean ± SD of three technical replicates. **m, q** One-way ANOVA with Tukey's multiple comparisons test. ***p<0.01. Source data are provided as a Source data file.

Supplementary Figure 2

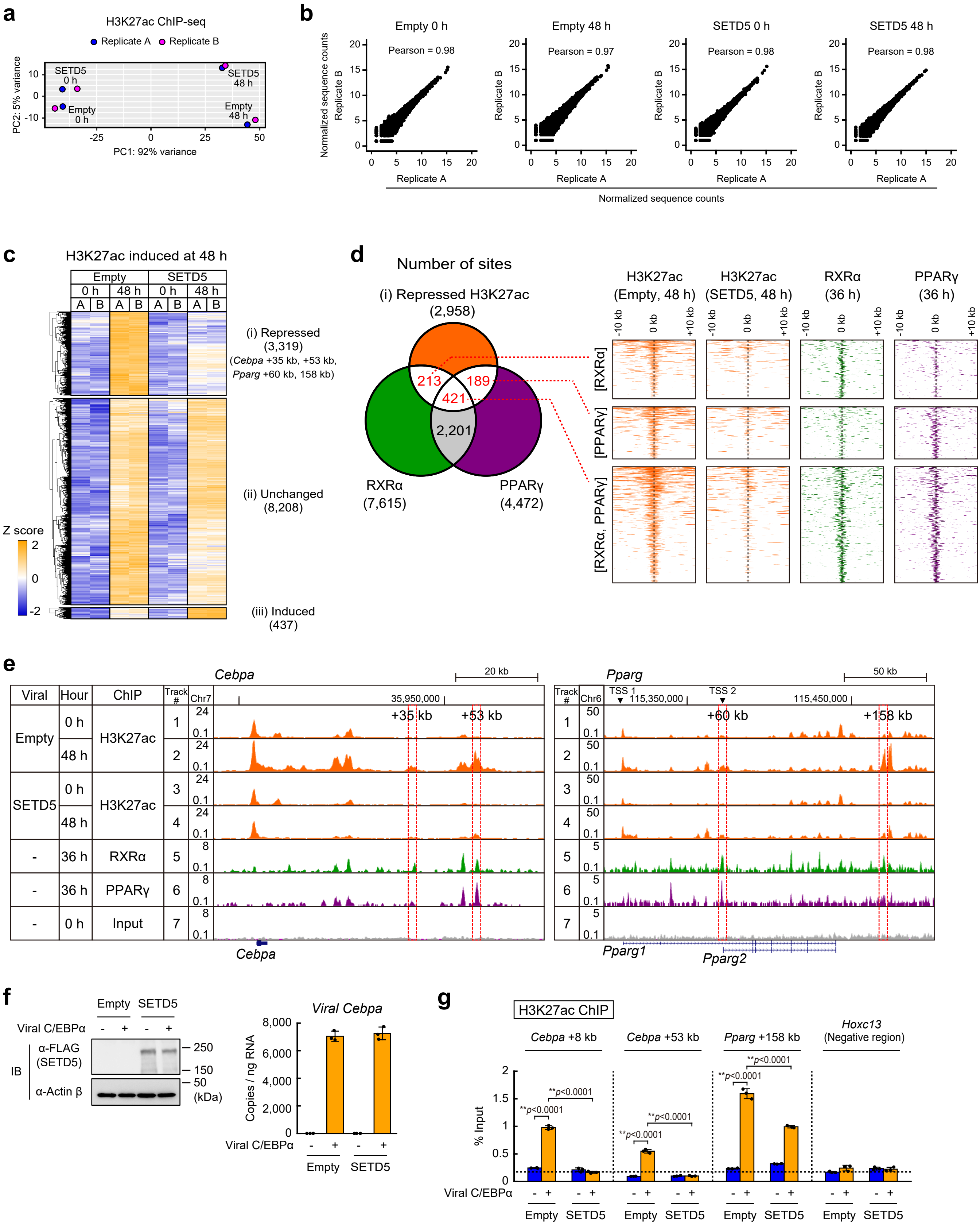

Supplementary Fig. 2 SETD5 inhibits H3K27 acetylation and enhancer activation of adipogenic genes. **a** Principal component (PC) analysis on H3K27ac ChIP-seq peaks in 3T3-L1 preadipocytes transduced with control empty virus and SETD5-V5. **b** Scatter plots showing the reproducibility of H3K27ac ChIP-seq enrichment at individual peaks between two biological replicates. **c** Statistical analysis of differentially regulated H3K27 acetylated regions using DeSeq2. H3K27 acetylation at 48 hrs was repressed in 3,319 regions (<0.5-fold) and induced in 437 regions (>2-fold) in SETD5 transduced cells compared to empty virus transduced cells. **d** Venn diagram and heatmap representation of differentiation-induced and H3K27ac enriched regions and RXRα and PPARγ binding regions. RXRα and PPARγ binding regions were identified using MACS program. Among differentiation-induced and SETD5-repressed H3K27ac enriched enhancer regions, 213 regions were bound by RXRα, 189 regions were bound by PPARγ, and 421 regions were bound by both. Heatmap shows ChIP-seq data for H3K27ac, RXRα, and PPARγ at a 20-kb region centered on H3K27ac enriched region. **e** Genome browser representation for H3K27ac, RXRα, and PPARγ on adipogenic genes. 3T3-L1 preadipocytes transduced with empty virus or SETD5 were subjected to ChIP-seq for H3K27ac at the indicated day of differentiation. Tracks 1-4 and 7 are the same data as Fig. 2e. **f** Retroviral expression of FLAG-tagged SETD5 and C/EBPα. Whole-cell lysate from 3T3-L1 preadipocytes transduced with the indicated retroviruses was subjected to immunoblot (IB) analysis (*left*). Equal loading of the proteins was confirmed by blotting with anti-actin β antibody. mRNA expression of retroviral *Cebpa* was quantified by qPCR (*right*). **g** ChIP-qPCR analysis of H3K27ac on *Cebpa* and *Pparg* genes in 3T3-L1 preadipocytes transduced with SETD5 and C/EBPα. 3T3-L1 preadipocytes transduced with the indicated retroviruses were subjected to ChIP-qPCR analysis of H3K27ac before differentiation induction. **d-e** ChIP-seq for RXRα, PPARγ, and input was from previously published papers [17] [13]. **f-g** Representative two independent experiments. Data are mean ± SD of three technical replicates. **g** One-way ANOVA with Tukey’s multiple comparisons test. ***p*<0.01. Source data are provided as a Source data file.

Supplementary Figure 3

a

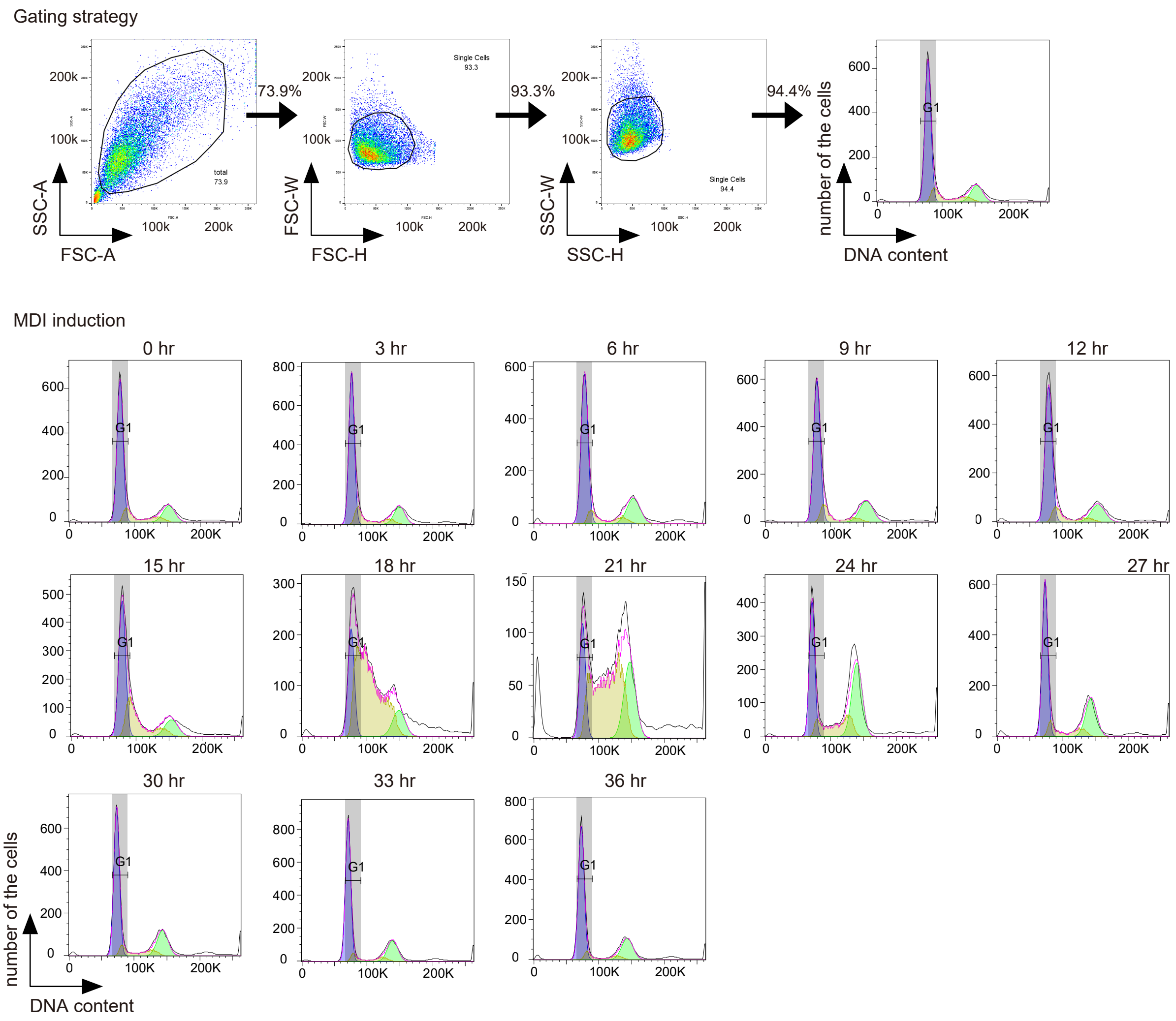

b

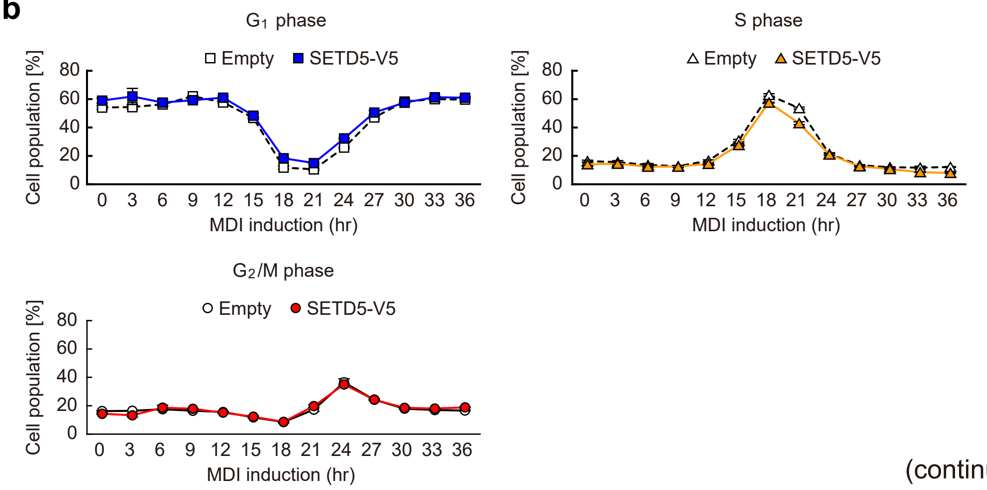

(continued on next page)

Supplementary Figure 3

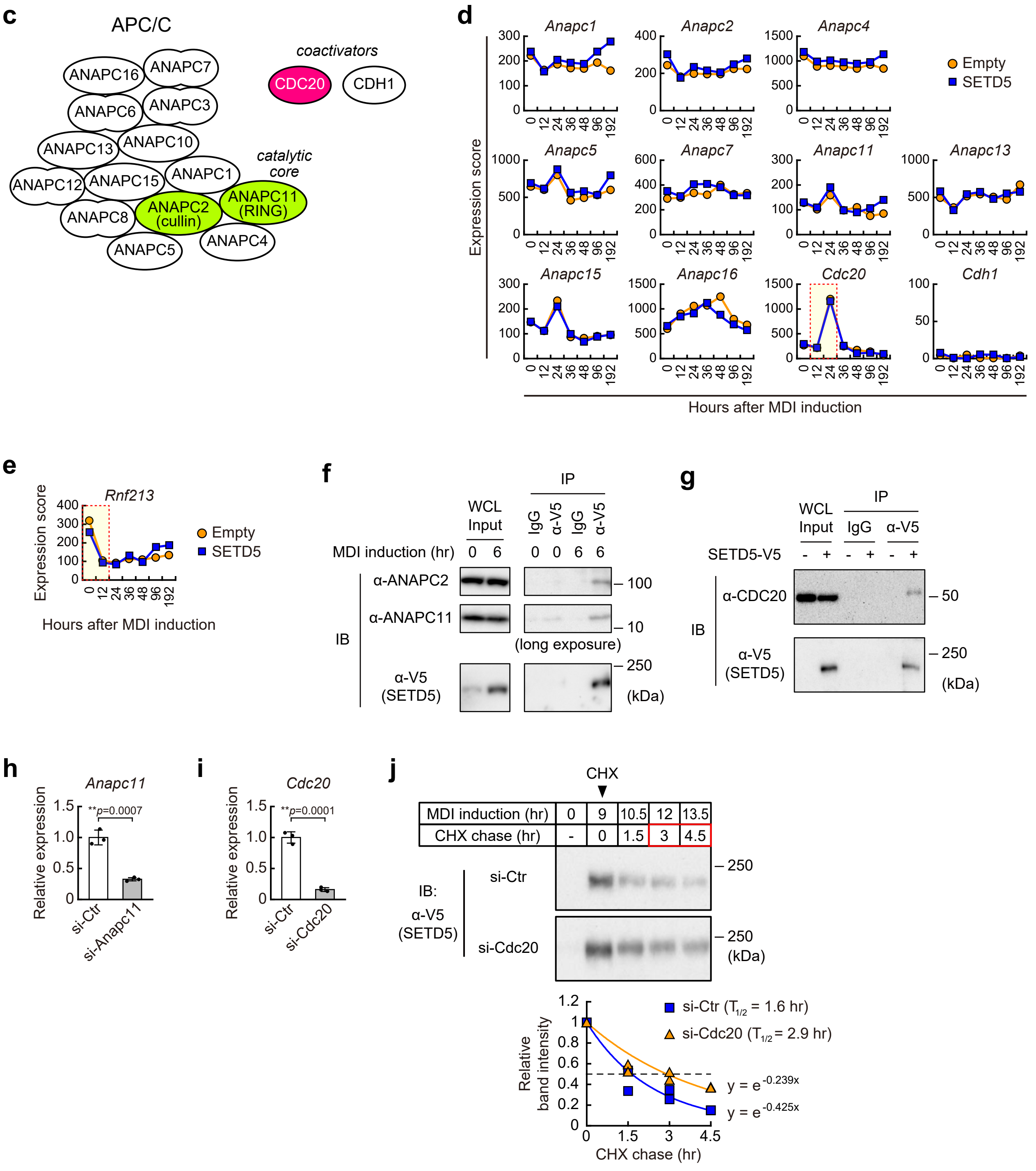

(continued from the previous page)

Supplementary Fig. 3 Transient stabilization and APC/C-mediated proteasomal degradation of SETD5 during the early adipogenesis. **a** Gating strategy for the analysis of cell cycle by flow cytometer was indicated in top panels. Debris was removed by FSC-A/SSC-A scatter gate. Then, doublets were excluded by FSC-H/FSC-W and SSC-H/SSC-W gates. Representative histograms of the DNA content of 3T3-L1 preadipocytes transduced with SETD5-V5 during adipogenesis were shown in the bottom panels. Blue indicates cells at G_1 phase; yellow indicates cells at S phase; green indicates cells at G_2/M phase. **b** Cell cycle analysis of 3T3-L1 preadipocytes transduced with empty virus or SETD5-V5. The ratios of the cells at G_1, S, or G_2/M phase of preadipocytes during differentiation are shown. Data for preadipocytes transduced with empty virus are the same data as Fig. 3c. **c** Schematic representation of APC/C and its coactivators. **d, e** Transcriptional changes of APC/C subunit genes (**d**) and *Rnf213* (**e**) in 3T3-L1 preadipocytes transduced with empty virus or SETD5 during adipogenesis. Cells were harvested at the indicated times of differentiation, and transcriptional analyses were carried out using a microarray. **f, g** Co-immunoprecipitation of SETD5 with ANAPC2, ANAPC11, and CDC20. Whole-cell lysate (WCL) from 3T3-L1 preadipocytes transduced with SETD5-V5 at the indicated time of differentiation was subjected to immunoprecipitation using anti-V5 antibody followed by immunoblot (IB) analysis using anti-ANAPC2, anti-ANAPC11, or anti-V5 antibody (**f**). 3T3-L1 preadipocytes transduced with SETD5-V5 were induced with MDI and then treated with MG-132 and whole-cell lysate was subjected to immunoprecipitation using anti-V5 antibody followed by immunoblot (IB) analysis using anti-CDC20 or anti-V5 antibody (**g**). **h, i** siRNA-mediated knockdown of ANAPC11 (**h**) and CDC20 (**i**) in 3T3-L1 preadipocytes transduced with SETD5-V5. *Anapc11* and *Cdc20* mRNA expression was quantified by qPCR. **j** Immunoblot showing SETD5-V5 stability by the cycloheximide chase assay. 3T3-L1 preadipocytes transduced with SETD5-V5 were transfected with control siRNA or siRNA targeted to *Cdc20* and induced for differentiation with MDI mixture. Preadipocytes were treated with cycloheximide (CHX) at 9 hrs after MDI induction and subjected to immunoblot analysis with anti-V5 at the indicated time of differentiation. The experiments were performed twice, and the representative immunoblot is shown. The graph shows regression analysis of SETD5-V5 protein stability in the above immunoblotting after CHX treatment from two independent experiments. Protein half-life ($T_{1/2}$) was calculated based on exponential decay curve fit to the average

data at each time point. **f-g** Representative of two independent experiments. **b, h-i** Data are mean ± SD of three technical replicates. **h-i** Unpaired two-tailed Student's *t* test. ***p*<0.01. Source data are provided as a Source data file.

Supplementary Figure 4

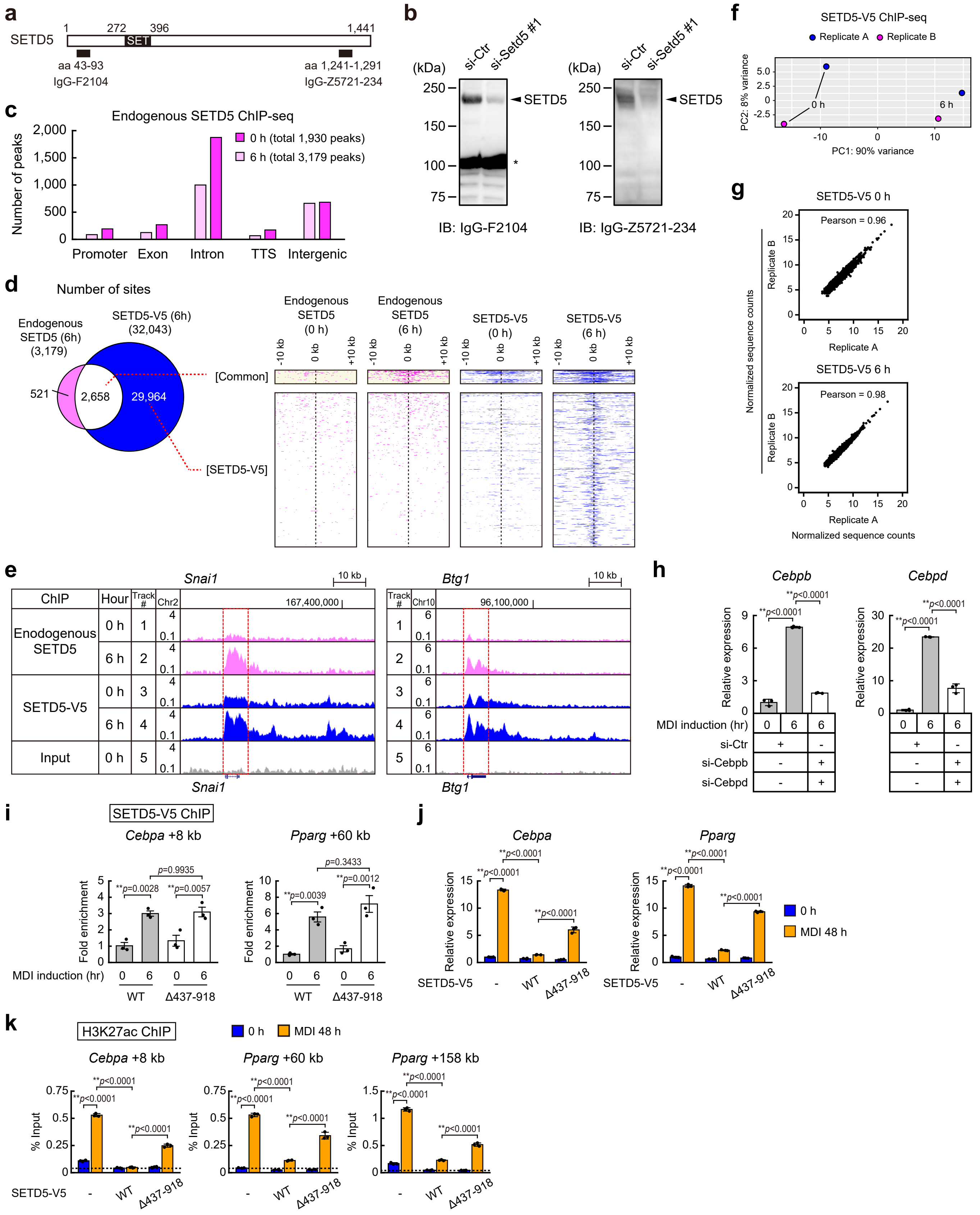

Supplementary Fig. 4 Genome-wide analysis of SETD5 binding sites during early adipogenesis. **a** Schematic representation of mouse SETD5 protein and fragments to develop anti-SETD5 antibodies. Mouse monoclonal immunoglobulin G (IgG)-Z5721-234 was developed by immunizing mice with recombinant SETD5 fragment (amino acids 1,241-1,291) expressed in Sf9 cells. IgG-F2104 was developed by immunizing mice with recombinant SETD5 fragment (amino acids 43-93). **b** Immunoblot analysis of endogenous SETD5 in 3T3-L1 preadipocytes. 3T3-L1 preadipocytes transfected with control siRNA or siRNA targeting to *Setd5* were subjected to immunoblot analysis using anti-SETD5 antibodies. Asterisk indicates nonspecific signals. **c** Genome-wide distribution of endogenous SETD5 before (0 h) and 6 hrs of differentiation in 3T3-L1 preadipocytes. TTS, transcription termination site. **d** Venn diagram (*left*) and heatmap (*right*) representation of genomic regions occupied by endogenous SETD5 and retrovirally expressed SETD5-V5. Endogenous SETD5 and SETD5-V5 binding regions were identified using SICER program. Among 32,043 SETD5-V5 binding regions at 6 hrs of differentiation, only 2,658 regions were identified as endogenous SETD5 binding regions by ChIP-seq using IgG-F2104. Heatmap analysis showing ChIP-seq data for endogenous SETD5 and SETD5-V5 at a 20-kb region centered on SETD5-V5 binding region. Data for SETD5-V5 are the same as Fig. 4b. **e** Genome browser representation for endogenous SETD5 on *Snai1* and *Btg1* genes in 3T3-L1 preadipocytes. 3T3-L1 preadipocytes were subjected to ChIP-seq for SETD5 using IgG-F2104 at the indicated time after differentiation induction. ChIP-seq for control input was from previously published paper [13]. **f** Principal component (PC) analysis on SETD5-V5 ChIP-seq peaks in 3T3-L1 preadipocytes transduced with SETD5-V5 at the indicated time after MDI induction. **g** Scatter plots showing the reproducibility of SETD5-V5 ChIP-seq enrichment at individual peaks between two biological replicates. **h** siRNA-mediated knockdown of C/EBPβ and C/EBPδ in 3T3-L1 preadipocytes transduced with SETD5-V5. *Cebpb* and *Cebpd* mRNA expression was quantified by qPCR. **i** ChIP-qPCR analysis wild-type and Δ437-918 SETD5-V5 on *Cebpa* and *Pparg* genes. 3T3-L1 preadipocytes transduced with wild-type and Δ437-918 SETD5-V5 were subjected to ChIP-qPCR analysis using anti-V5 antibody at the indicated time after differentiation induction. **j** Transcriptional changes of *Cebpa* and *Pparg* in wild-type and Δ437-918 SETD5-V5 transduced 3T3-L1 preadipocytes during early adipogenesis. mRNA expression was quantified by qPCR. **k** ChIP-qPCR

analysis of H3K27ac on *Cebpa* and *Pparg* genes during adipogenesis. 3T3-L1 preadipocytes transduced with wild-type and Δ437-918 SETD5-V5 were subjected to ChIP-qPCR analysis of H3K27ac at the indicated time after MDI induction of differentiation. **b, h, j-k** Representative of two independent experiments. **h, j-k** Data are mean ± SD of three technical replicates. **i** Data are mean ± SEM of three independent experiments. **h-k** One-way ANOVA with Tukey's multiple comparisons test. $^{**}p<0.01$. Source data are provided as a Source data file.

Supplementary Figure 5

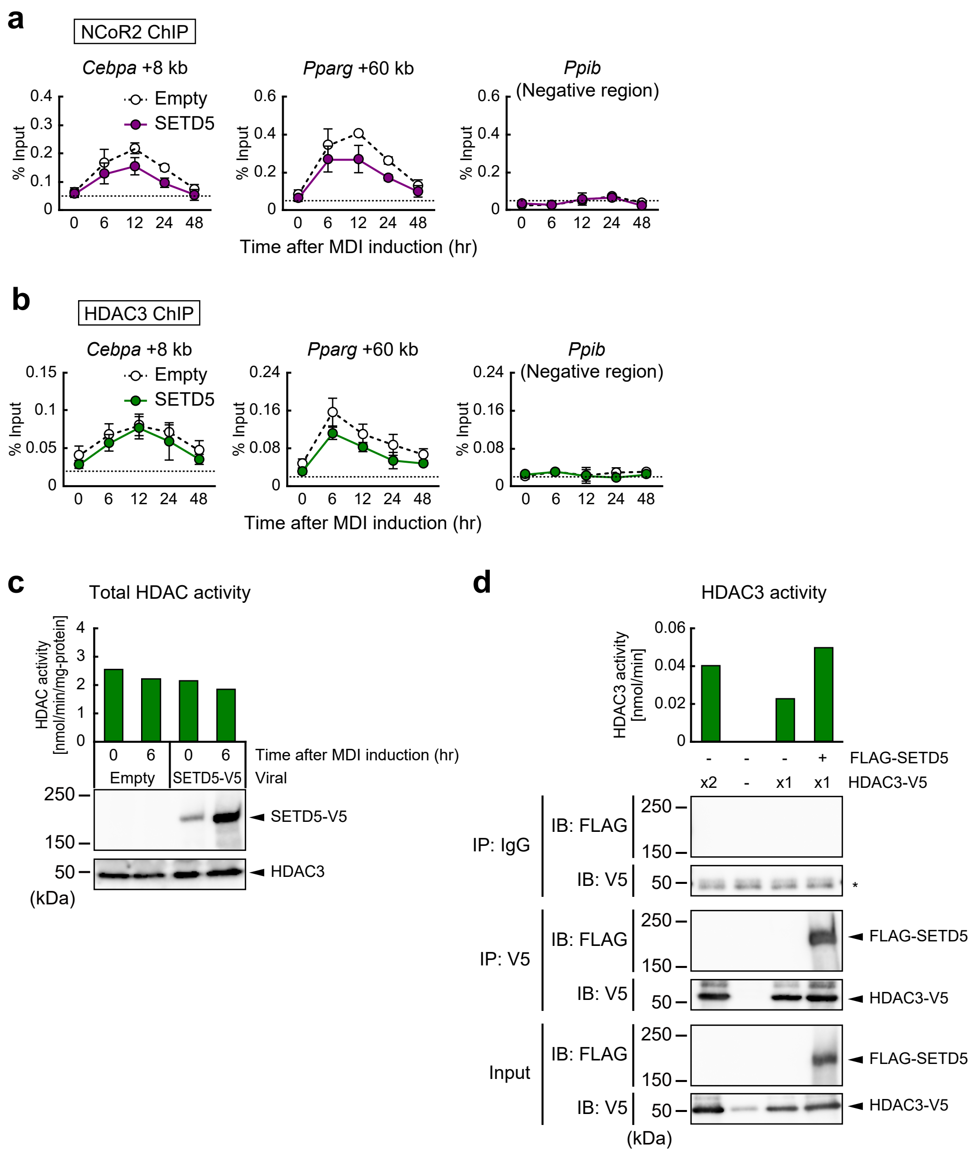

Supplementary Fig. 5 Effects of SETD5 on NCoR and HDAC recruitment and HDAC activity. **a, b** ChIP-qPCR analyses of NCoR2 (**a**) and HDAC3 (**b**) on *Cebpa* and *Pparg* genes during the early adipogenesis. 3T3-L1 preadipocytes transduced with control empty virus or SETD5-V5 were subjected to ChIP-qPCR analyses using anti-NCoR2 or anti-HDAC3 antibody. Data are mean ± SEM of (0, 48 hr *n*=5; 6 hr of NCoR2 *n*=4; 6 hr of HDAC3 *n*=5; 12, 24 hr *n*=3; independent experiments). Data for preadipocytes transduced with empty virus are the same data as Fig. 5c and 5d. **c** Effect of SETD5 on total HDAC activity. Nuclear proteins from 3T3-L1 preadipocytes transduced with control empty virus or SETD5-V5 was subjected to in vitro assay for total HDAC activity. Expression of SETD5-V5 and HDAC3 was confirmed by immunoblot analysis using anti-V5 and anti-HDAC3 antibodies. **d** Effect of SETD5 on HDAC3 activity. HEK293 cells were co-transfected with plasmids encoding FLAG-SETD5 and HDAC3-V5. Nuclear proteins were prepared and subjected to immunoprecipitation using control mouse IgG or anti-V5 antibody. Immunoprecipitates were subjected to in vitro assay for HDAC activity. Co-transfection of FLAG-SETD5 somehow increased protein level as well as activity of HDAC3-V5 by ~2-fold (comparison of lanes 3 and 4). Cells transfected with twice more plasmid encoding HDAC3-V5 alone (lane 1) showed similar protein level and activity of HDAC3 to co-transfected cells (lane 4). Asterisk indicates control mouse IgG signals. **c-d** Representative of two independent experiments. Source data are provided as a Source data file.

Supplementary Figure 6

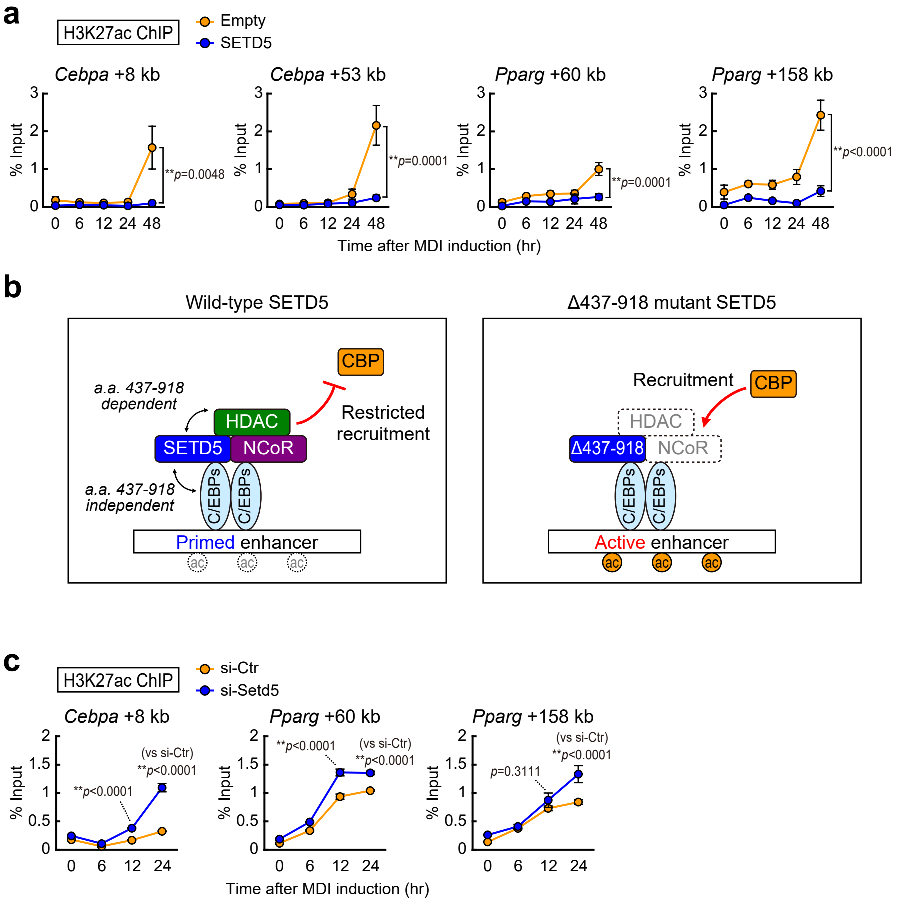

Supplementary Fig. 6 SETD5-NCoR-HDAC3 complex restricts CBP recruitment to primed enhancers. **a** ChIP-qPCR analysis of H3K27ac on *Cebpa* and *Pparg* genes during the early adipogenesis. 3T3-L1 preadipocytes transduced with control empty virus or SETD5 were subjected to ChIP-qPCR analyses using anti-H3K27ac antibody. Data are mean ± SEM (0, 6, 12, 24 hr n=3; 48 hr n=5; independent experiments). **b** Schematic model for regulation of CBP recruitment by SETD5-NCoR-HDAC3 complex. Wild-type SETD5 forms a complex with NCoR and HDAC3 and is recruited to primed enhancers via C/EBP transcription factors to restrict CBP recruitment (*left*). The Δ437-918 mutant SETD5 is recruited to primed enhancers but does not form a complex with NCoR and HDAC3 and does not restrict CBP recruitment (*right*). **c** ChIP-qPCR analysis of H3K27ac on *Cebpa* and *Pparg* genes in SETD5-knocked down preadipocytes. 3T3-L1 preadipocytes transfected with control siRNA or siRNA targeting to *Setd5* were subjected to ChIP-qPCR analyses using anti-H3K27ac antibody. Representative of two independent experiments. Data are mean ± SD of three technical replicates. **a, c** One-way ANOVA with Tukey's multiple comparisons test. **p<0.01. Source data are provided as a Source data file.

Supplementary References

1. Kimura H, Hayashi-Takanaka Y, Goto Y, Takizawa N, Nozaki N. The organization of histone H3 modifications as revealed by a panel of specific monoclonal antibodies. *Cell structure and function* **33**, 61-73 (2008).

2. Abe Y, *et al.* JMJD1A is a signal-sensing scaffold that regulates acute chromatin dynamics via SWI/SNF association for thermogenesis. *Nature Communications* **6**, 7052 (2015).

3. Zhang Y, *et al.* Model-based analysis of ChIP-Seq (MACS). *Genome biology* **9**, R137 (2008).

4. Zang C, Schones DE, Zeng C, Cui K, Zhao K, Peng W. A clustering approach for identification of enriched domains from histone modification ChIP-Seq data. *Bioinformatics* **25**, 1952-1958 (2009).

5. Xu S, Grullon S, Ge K, Peng W. Spatial clustering for identification of ChIP-enriched regions (SICER) to map regions of histone methylation patterns in embryonic stem cells. *Methods Mol Biol* **1150**, 97-111 (2014).

6. Huang DW, Sherman BT, Lempicki RA. Systematic and integrative analysis of large gene lists using DAVID bioinformatics resources. *Nature protocols* **4**, 44-57 (2008).

7. Heinz S, *et al.* Simple combinations of lineage-determining transcription factors prime cis-regulatory elements required for macrophage and B cell identities. *Mol Cell* **38**, 576-589 (2010).

8. Langmead B, Salzberg SL. Fast gapped-read alignment with Bowtie 2. *Nat Methods* **9**, 357-359 (2012).

9. Bolger AM, Lohse M, Usadel B. Trimmomatic: a flexible trimmer for Illumina sequence data. *Bioinformatics* **30**, 2114-2120 (2014).

10. Li H, *et al.* The Sequence Alignment/Map format and SAMtools. *Bioinformatics*

25, 2078-2079 (2009).

11. Quinlan AR, Hall IM. BEDTools: a flexible suite of utilities for comparing genomic features. *Bioinformatics* **26**, 841-842 (2010).

12. Love MI, Huber W, Anders S. Moderated estimation of fold change and dispersion for RNA-seq data with DESeq2. *Genome Biol* **15**, 550 (2014).

13. Matsumura Y, *et al.* H3K4/H3K9me3 bivalent chromatin domains targeted by lineage-specific DNA methylation pauses adipocyte differentiation. *Molecular cell* **60**, 584-596 (2015).

14. Siersbæk R, *et al.* Transcription factor cooperativity in early adipogenic hotspots and super-enhancers. *Cell Rep* **7**, 1443-1455 (2014).

15. Siersbæk R, *et al.* Dynamic Rewiring of Promoter-Anchored Chromatin Loops during Adipocyte Differentiation. *Mol Cell* **66**, 420-435 e425 (2017).

16. Gao T, Qian J. EnhancerAtlas 2.0: an updated resource with enhancer annotation in 586 tissue/cell types across nine species. *Nucleic Acids Res* **48**, D58-D64 (2020).

17. Waki H, *et al.* Global mapping of cell type-specific open chromatin by FAIRE-seq reveals the regulatory role of the NFI family in adipocyte differentiation. *PLoS Genet* **7**, e1002311 (2011).
