# Supplementary material for: Spatiotemporal dynamics of SETD5-containing NCoR–HDAC3 complex determines enhancer activation for adipogenesis
Source: Nat Commun. 2021 Dec 2;12:7045. doi: 10.1038/s41467-021-27321-5 (PMC8639990; doi:10.1038/s41467-021-27321-5)
Supplement: Supplementary file 3 — Description of additional Supplementary File [file 41467_2021_27321_MOESM3_ESM.pdf]

Description of additional supplementary data files

Supplementary Data 1: List of identified proteins by proteomics.
