# Supplementary material for: Spatiotemporal dynamics of SETD5-containing NCoR–HDAC3 complex determines enhancer activation for adipogenesis
Source: Nat Commun. 2021 Dec 2;12:7045. doi: 10.1038/s41467-021-27321-5 (PMC8639990; doi:10.1038/s41467-021-27321-5)
Supplement: Supplementary file 5 — Reporting Summary [file 41467_2021_27321_MOESM5_ESM.pdf]

nature portfolio

Corresponding author(s): Juro Sakai

Last updated by author(s): Oct 25, 2021

Reporting Summary

Nature Portfolio wishes to improve the reproducibility of the work that we publish. This form provides structure for consistency and transparency in reporting. For further information on Nature Portfolio policies, see our Editorial Policies and the Editorial Policy Checklist.

Statistics

For all statistical analyses, confirm that the following items are present in the figure legend, table legend, main text, or Methods section.

n/a	Confirmed	
☐	☒	The exact sample size (*n*) for each experimental group/condition, given as a discrete number and unit of measurement
☐	☒	A statement on whether measurements were taken from distinct samples or whether the same sample was measured repeatedly
☐	☒	The statistical test(s) used AND whether they are one- or two-sided<br>*Only common tests should be described solely by name; describe more complex techniques in the Methods section.*
☒	☐	A description of all covariates tested
☒	☐	A description of any assumptions or corrections, such as tests of normality and adjustment for multiple comparisons
☐	☒	A full description of the statistical parameters including central tendency (e.g. means) or other basic estimates (e.g. regression coefficient) AND variation (e.g. standard deviation) or associated estimates of uncertainty (e.g. confidence intervals)
☐	☒	For null hypothesis testing, the test statistic (e.g. *F*, *t*, *r*) with confidence intervals, effect sizes, degrees of freedom and *P* value noted<br>*Give P values as exact values whenever suitable.*
☒	☐	For Bayesian analysis, information on the choice of priors and Markov chain Monte Carlo settings
☒	☐	For hierarchical and complex designs, identification of the appropriate level for tests and full reporting of outcomes
☐	☒	Estimates of effect sizes (e.g. Cohen's *d*, Pearson's *r*), indicating how they were calculated

Our web collection on statistics for biologists contains articles on many of the points above.

Software and code

Policy information about availability of computer code

Data collection	Affymetrix GeneChip scanner 3000 for microarray. Illumina Sequencing Control Software v2.10.17 and HiSeq Control Software v2.2.58 for ChIP-seq. Olympus cellSens standard 1.17 for microscopy images. Progenesis LC/MS v2.6 for proteomics.
Data analysis	Affymetrix GeneChip Analysis Suite software version 5.0 for microarray analysis. MACS1.4.2, MACS2.2.7.1, SICER1.1, SICER2-1.0.2, DAVID6.7, Homer4.10.4, CASSAVA1.8.2, Bowtie2.3.4.3, Trimmomatic0.39, Samtools1.9, Bedtools2.27.1, and DEseq2.1.30.1 for ChIP-seq analysis. ImageJ1.53k for quantification of immunoblot. FlowJo v10 for flow cytometry analysis. Mascot v2.3 for proteomics.

For manuscripts utilizing custom algorithms or software that are central to the research but not yet described in published literature, software must be made available to editors and reviewers. We strongly encourage code deposition in a community repository (e.g. GitHub). See the Nature Portfolio guidelines for submitting code & software for further information.

Data

Policy information about availability of data

All manuscripts must include a data availability statement. This statement should provide the following information, where applicable:

- Accession codes, unique identifiers, or web links for publicly available datasets
- A description of any restrictions on data availability
- For clinical datasets or third party data, please ensure that the statement adheres to our policy

Gene expression microarray data and ChIP-seq data for H3K27ac, SETD5-V5, H3K4me1, and SETD5 were deposited in the Gene Expression Omnibus (GEO) database with accession numbers GSE183849 (https://www.ncbi.nlm.nih.gov/geo/query/acc.cgi?acc=GSE183849). Other ChIP-seq data were already published and deposited in the GEO database GSE73434 (https://www.ncbi.nlm.nih.gov/geo/query/acc.cgi?acc=GSE73434) or the DNA data bank of Japan (DRA000378). Previously reported ChIP-seq data for H3K27ac, C/EBPβ, C/EBPδ, HDAC3, NCoR, and p300 are available in the GEO database GSE 27826 (https://www.ncbi.nlm.nih.gov/geo/query/

acc.cgi?acc=GSE27826), GSE95533 (https://www.ncbi.nlm.nih.gov/geo/query/acc.cgi?acc=GSE95533), and GSE56872 (https://www.ncbi.nlm.nih.gov/geo/query/acc.cgi?acc=GSE56872). Proteomics data were deposited in ProteomeXchange with the accession number PXD029279 (http://proteomecentral.proteomexchange.org/cgi/GetDataset?ID=PXD029279).

Field-specific reporting

Please select the one below that is the best fit for your research. If you are not sure, read the appropriate sections before making your selection.

- [x] Life sciences
- [ ] Behavioural & social sciences
- [ ] Ecological, evolutionary & environmental sciences

For a reference copy of the document with all sections, see nature.com/documents/nr-reporting-summary-flat.pdf

Life sciences study design

All studies must disclose on these points even when the disclosure is negative.

Sample size	Sample sizes were selected based on previously studies with similar type of experiments (PMID:25948511, 26590716, 29674659).
Data exclusions	No data were excluded.
Replication	Experiments were repeated at least twice with the independent biological samples using similar experimental conditions or otherwise mentioned in the respective figure legends, main text or methods section.
Randomization	All experiments were conducted using randomly assigned animals and cells culture populations.
Blinding	For cell culture and molecular experiments (RT-qPCR, ChIP-qPCR, and immunoblot), blinding was not applicable. For transplantation experiments, investigators were blinded during tissue staining and evaluation of the data.

Reporting for specific materials, systems and methods

We require information from authors about some types of materials, experimental systems and methods used in many studies. Here, indicate whether each material, system or method listed is relevant to your study. If you are not sure if a list item applies to your research, read the appropriate section before selecting a response.

Materials & experimental systems

n/a	Involved in the study	
[ ]	[x]	Antibodies
[ ]	[x]	Eukaryotic cell lines
[x]	[ ]	Palaeontology and archaeology
[ ]	[x]	Animals and other organisms
[x]	[ ]	Human research participants
[x]	[ ]	Clinical data
[x]	[ ]	Dual use research of concern

Methods

n/a	Involved in the study	
[ ]	[x]	ChIP-seq
[ ]	[x]	Flow cytometry
[x]	[ ]	MRI-based neuroimaging

Antibodies

Antibodies used

Following are the antibodies used in this study:
1. Anti-H3K27ac mouse mAb 9E2H9 (Institute of Innovative Research, Tokyo Institute of Technology, Japan)
2. Anti-mouse SETD5 mouse mAb IgG-F2104 (developed as described in Methods)
3. Anti-mouse SETD5 mouse mAb IgG-Z5721-234 (developed as described in Methods)
4. Anti-TBP mouse mAb (Novus Biologicals, NB500-700, clone 1TBP18)
5. Anti-V5 mouse mAb (Thermo Scientific, R960-25)
6. Anti-Histone H3 rabbit pAb (Abcam, ab1791)
7. Anti-HDAC1 mouse mAb (Santa Cruz Biotechnology, sc-8410, clone H-11)
8. Anti-HDAC3 rabbit pAb (Abcam, ab7030)
9. Anti-NCoR2 rabbit pAb (Abcam, ab5802)
10. Anti-C/EBPβ rabbit pAb (Santa Cruz Biotechnology, sc-150x)
11. Anti-C/EBPβ mouse mAb (Santa Cruz Biotechnology, sc-7962, clone H-7)
12. Anti-CBP rabbit mAb (Cell Signaling Technology, 7425, clone D9B6)
13. Anti-H3K4me1 rabbit pAb (Abcam, ab8895)
14. Anti-H3K4me3 rabbit pAb (Merck Millipore, 07-473)
15. Anti-Multi ubiquitin mouse mAb (Medical and Biological Laboratories, D058-3, clone FK2)
16. Anti-ANAPC2 rabbit pAb (Cell Signaling Technology, 12301)
17. Anti-ANAPC11 rabbit mAb (Cell Signaling Technology, 14090, clone D1E7Q)

18. Anti-CDC20 rabbit mAb (Cell Signaling Technology, 14866, clone D6C2Q)
19. Anti-Actin β mouse mAb (Sigma-Aldrich, A5441, clone AC15)
20. Anti-FLAG mouse mAb (Sigma-Aldrich, F3165, clone M2)
21. Anti-Perilipin-1 rabbit mAb (Cell Signaling Technology, 9349, clone D1D8)
22. Anti-mouse IgG-HRP (Sigma-Aldrich, A4416)
23. Anti-rabbit IgG-HRP (Sigma-Aldrich, A0545)

Validation

The validation of antibodies used was performed by the individual companies and publications.
1. Anti-H3K27ac mouse mAb 9E2H9: Specificity was validated by ELISA, ChIP, WB, and IF (Kimura H. et al., Cell Struct. Funct., 2008, 33, 61-73, PMID: 18227620).
2. Anti-mouse SETD5 mouse mAb IgG-F2104: Specificity was validated by immunoblot in this manuscript as shown in Supplementary Fig. 4a, b.
3. Anti-mouse SETD5 mouse mAb IgG-Z5721-234: Specificity was validated by immunoblot in this manuscript as shown in Supplementary Fig. 4a, b.
4. Anti-TBP mouse mAb - Manufacturer's statement: 'This antibody recognizes an epitope within amino acid residues 1-20 of human, mouse, and rat TBP. Applications: Western Blot, ELISA, Gel Super Shift Assays, Immnuoprecipitation.' (https://www.novusbio.com/products/tata-binding-protein-tbp-antibody-1tbp18_nb500-700)
5. Anti-V5 mouse mAb -Manufacturer's statement: 'R960-25 recognizes amino acid sequence: -Gly-Lys-Pro-Ile-Pro-Asn-Pro-Leu-Leu-Gly-Leu-Asp-Ser-Thr-. This antibody is functionally tested against 20 ng of an E. coli expressed fusion protein containing a V5 epitope using a chemiluminescent substrate at a 1 minute exposure. Applications; WB, IHC, Flow cytometry, ELISA, IP, ChIP.' (https://www.thermofisher.com/antibody/product/V5-Tag-Antibody-Monoclonal/R960-25)
6. Anti-Histone H3 rabbit pAb -Manufactrer's statement: 'Rabbit polyclonal to Histone H3 - Nuclear Marker and ChIP Grade. Suitable for: ICC, IHC-P, ChIP, IP, WB. Reacts with: Mouse, Rat, Human, Saccharomyces cerevisiae, Xenopus laevis, Arabidopsis thaliana, Drosophila melanogaster, Indian muntjac, Schizosaccharomyces pombe. Isotype: IgG.' (https://www.abcam.co.jp/histone-h3-antibody-nuclear-marker-and-chip-grade-ab1791.html)
7. Anti-HDAC1 mouse mAb -Manufacturer's statement: 'Histone Deacetylase 1 (HDAC1) Antibody (H-11) is a mouse monoclonal IgG1 κ, cited in 88 publications, provided at 200 µg/ml. Raised against amino acids 432-482 mapping at the C-terminus of HDAC1 of human origin. Anti-Histone Deacetylase 1 (HDAC1) Antibody (H-11) is recommended for detection of HDAC1 of mouse, rat and human origin by WB, IP, IF, FCM and ELISA.' (https://www.scbt.com/p/hdac1-antibody-h-11?productCanUrl=hdac1-antibody-h-11&_requestid=465246)
8. Anti-HDAC3 rabbit pAb -Manufacturer's statement: 'Rabbit polyclonal to HDAC3. Suitable for: ICC, ICC/IF, IHC-P, ChIP, WB, Dot blot, IP. Reacts with: Mouse, Rat, Human, Monkey, African green monkey, Chinese hamster. Isotype: IgG.' (https://www.abcam.co.jp/hdac3-antibody-ab7030.html)
9. Anti-NCoR2 rabbit pAb -Manufacturer's statement: 'Rabbit polyclonal to NCOR2/SMRT. Suitable for: ICC/IF, IHC-P. Reacts with: Mouse, Human. Isotype: IgG.' (https://www.abcam.co.jp/ncor2smrt-antibody-ab5802.html)
10. Anti-C/EBPβ rabbit pAb -Manufacturer's statement: 'C/EBP β Antibody (C-19) is a rabbit polyclonal IgG; 200 µg/ml. Epitope mapping at the C-terminus of C/EBP β of rat origin.' (https://www.scbt.com/p/c-ebp-beta-antibody-c-19?productCanUrl=c-ebp-beta-antibody-c-19&_requestid=467688) Also validated to react with C/EBPβ of mouse origin in IP and ChIP in this manuscript (Fig. 4f) and other publications (Sierbæk R. et al., EMBO J., 2011, 30, 1549-1572, PMID: 21427703; Abe Y. et al., Nat. Commun., 2015, 6, 7052, PMID: 25948511).
11. Anti-C/EBPβ mouse mAb -Manufacturer's statement: 'Anti-C/EBP β Antibody (H-7) is a mouse monoclonal IgG2a κ C/EBP β antibody, cited in 214 publications, provided at 200 µg/ml. Raised against amino acids 199-345 of C/EBP β of human origin. Anti-C/EBP beta Antibody (H-7) is recommended for detection of C/EBP β of mouse, rat and human origin by WB, IP, IF and IHC(P).' (https://www.scbt.com/p/c-ebp-beta-antibody-h-7?productCanUrl=c-ebp-beta-antibody-h-7&_requestid=468460)
12. Anti-CBP rabbit mAb -Manufacturer's statement: 'CBP (D9B6) Rabbit mAb recognizes endogenous levels of total CBP protein. This antibody also shows some cross-reactivity with p300 protein. Species Reactivity: Human, Mouse, Rat, Monkey. Application: WB, IP, ChIP.'(https://www.cellsignal.com/products/primary-antibodies/cbp-d9b6-rabbit-mab/7425?country=USA)
13. Anti-H3K4me1 rabbit pAb -Manufacturer's statement: 'Rabbit polyclonal to Histone H3 (mono methyl K4) - ChIP Grade. Suitable for: ICC, ChIP, WB, IHC-P. Reacts with: Mouse, Rat, Cow, Human. Isotype: IgG.' (https://www.abcam.co.jp/histone-h3-mono-methyl-k4-antibody-chip-grade-ab8895.html)
14. Anti-H3K4me3 rabbit pAb -Manufacturer's statement: 'Anti-trimethyl-Histone H3 (Lys4) Antibody is a rabbit polyclonal antibody for detection of Histone H3 trimethylated at lysine 4. Also known as Anti-H3K4me3, this highly specific and well published antibody has been validated in ChIP, DB, WB, PIA, ChIP-seq.' (https://www.merckmillipore.com/JP/ja/product/Anti-trimethyl-Histone-H3-Lys4-Antibody,MM_NF-07-473)
15. Anti-Multi ubiquitin mouse mAb -Manufacturer's statement: 'Clone FK2 has been reported to recognize K29-, K48-, K63-linked poly ubiquitinylated and mono ubiquitinylated proteins but not free ubiquitin (PMID:18757370, 19237541).' (https://www.mblbio.com/bio/g/dtl/A/index.html?pcd=D058-3)
16. Anti-ANAPC2 rabbit mAb -Manufacturer's statement: 'Specificity/Sensitivity: APC2 Antibody recognizes endogenous levels of total APC2 protein. Species Reactivity: Human, Mouse, Rat, Monkey. Application: WB, IP.' (https://www.cellsignal.com/products/primary-antibodies/apc2-antibody/12301?country=USA)
17. Anti-ANAPC11 rabbit mAb -Manufacturer's statement: 'Specificity/Sensitivity: APC11 (D1E7Q) Rabbit mAb recognizes endogenous levels of total APC11 protein. This antibody does not cross-react with either RBX1 or RBX2. Species Reactivity: Human, Mouse, Rat, Monkey. Application: WB, IP.' (https://www.cellsignal.com/products/primary-antibodies/apc11-d1e7q-rabbit-mab/14090?country=USA)
18. Anti-CDC20 rabbit mAb -Manufacturer's statement: 'Specificity/Sensitivity: CDC20 (D6C2Q) Rabbit mAb recognizes endogenous levels of total CDC20 protein. This antibody does not cross-react with FZR1 protein. Species Reactivity: Human, Mouse, Rat, Monkey. Application: WB, IP.' (https://www.cellsignal.com/products/primary-antibodies/cdc20-d6c2q-rabbit-mab/14866?country=USA)
19. Anti-Actin β mouse mAb -Manufacturer's statement: 'Anti-β-Actin antibody, Mouse Monoclonal 1, 2 recognizes an epitope located on the N-terminal end of the β-isoform of actin. Monoclonal mouse anti-actin was used as a loading control for western blot analysis of rat liver protein lysates. The antibody has also been used for western blot at 0.5-1 µg/mL using cell extract of human foreskin fibroblasts or chicken fibroblasts. Species reactivity: pig, Hirudo medicinalis, bovine, rat, canine, feline, human, rabbit, carp, mouse, guinea pig, chicken, sheep.' (https://www.sigmaaldrich.com/US/en/product/sigma/a1978)
20. Anti-Perilipin-1 rabbit mAb -Manufacturer's statement: 'Specificity/Sensitivity: Perilipin-1 (D1D8) XP® Rabbit mAb detects

endogenous levels of total perilipin-1 protein. Species Reactivity: Human, Mouse. Application: WB, IP, IHC, IF.' (https://www.cellsignal.com/products/primary-antibodies/perilipin-1-d1d8-xp-rabbit-mab/9349?country=USA)
21. Anti-FLAG mouse mAb -Manufacturer's statement: 'Anti Flag M2 antibody is used for the detection of Flag fusion proteins. Application: immunoblotting, immunoprecipitation, immunocytochemistry, immunofluorescence, ELISA, EIA, chromatin immunoprecipitation, electron microscopy, flow cytometry, supershift assays.' (https://www.sigmaaldrich.com/US/en/product/sigma/f3165)
22. Anti-mouse IgG-HRP -Manufacturer's statement: 'Anti-Mouse IgG (whole molecule)-Peroxidase antibody is suitable for use in immunoblot. Species reactivity: mouse.' (https://www.sigmaaldrich.com/US/en/product/sigma/a4416)
23. Anti-rabbit IgG-HRP -Manufacturer's statement: 'Anti-Rabbit IgG (whole molecule)–Peroxidase antibody has been used in western blotting , immunofluorescence staining , immunochemistry and immunoprecipitations. Species reactivity: rabbit.' (https://www.sigmaaldrich.com/US/en/product/sigma/a0545)

Eukaryotic cell lines

Policy information about cell lines

Cell line source(s): 3T3-L1 (ATCC); HEK293 (ATCC); Sf9 (ATCC); Plat-E (Cosmo Bio Co. Ltd.)

Authentication: 3T3-L1 cells were validated by proteomics and ChIP-seq as mouse cell line (i.e. mouse-unique sequences detected). HEK293, Sf9, and Plat-E cells were not authenticated.

Mycoplasma contamination: Four cell lines are tested for mycoplasma contamination and found to be negative.

Commonly misidentified lines (See ICLAC register): No misidentified cell lines were used in this study.

Animals and other organisms

Policy information about studies involving animals; ARRIVE guidelines recommended for reporting animal research

Laboratory animals: Male nude mice (BALB/cSlc-nu/nu, purchased from Japan SLC, Inc) were fed standard chow (CE-2, CLEA Japan Inc.) ad libitum in a temperature- and humidity-controlled environment with a 12 hr light/12 hr dark cycle (08:00-20:00) at constant temperature (23ºC). Transplantation experiments were performed at 6 weeks of age.

Wild animals: No wild animals were used in this study.

Field-collected samples: No field collected samples were used.

Ethics oversight: All animal studies were approved by the Animal Care and Use Committee of Tohoku University.

Note that full information on the approval of the study protocol must also be provided in the manuscript.

ChIP-seq

Data deposition

- [x] Confirm that both raw and final processed data have been deposited in a public database such as GEO.
- [x] Confirm that you have deposited or provided access to graph files (e.g. BED files) for the called peaks.

Data access links
May remain private before publication.
ChIP-seq data were deposited in the Gene Expression Omnibus (GEO) database with accession numbers GSE183849.
https://www.ncbi.nlm.nih.gov/geo/query/acc.cgi?acc=GSE183849
The link is private until publication of the manuscript.

Files in database submission: FASTQ, bigwig, and graph files were deposited.

Genome browser session (e.g. UCSC):
Reviewer access links of Genome browser session are as follow:
https://genome.ucsc.edu/s/Yoshihiro%20Matsumura/mm9_Cebpa
https://genome.ucsc.edu/s/Yoshihiro%20Matsumura/mm9_Pparg

Methodology

Replicates: H3K27ac and SETD5-V5 ChIP-seq was done in two biological replicates. H3K4me1 and endogenous SETD5 ChIP-seq was done in one replicate.

Sequencing depth:
Number of total reads in each sample:
L1_E_D0_H3K27ac_RepA: 17,043,790
L1_E_D2_H3K27ac_RepA: 16,364,976
L1_SETD5_D0_H3K27ac_RepA: 25,915,409
L1_SETD5_D2_H3K27ac_RepA: 18,159,083
L1_E_D0_H3K27ac_RepB: 17,936,304

L1_E_D2_H3K27ac_RepB: 17,345,307
L1_SETD5_D0_H3K27ac_RepB: 19,442,128
L1_SETD5_D2_H3K27ac_RepB: 20,734,244
L1_0h_SETD5-V5_RepA: 43,312,378
L1_6h_SETD5-V5_RepA: 60,284,005
L1_0h_SETD5-V5_RepB: 56,211,074
L1_6h_SETD5-V5_RepB: 60,167,657
L1_D0_H3K4me1: 31,875,156
L1_D2_H3K4me1: 31,629,740
L1_0h_SETD5: 35,051,923
L1_6h_SETD5: 30,014,330

Antibodies

Anti-H3K27ac mouse mAb 9E2H9 (PMID: 18227620)
Anti-V5 mouse mAb (Thermo Scientific, R960-25)
Anti-H3K4me1 rabbit pAb (Abcam, ab8895)
Anti-mouse SETD5 mouse mAb IgG-F2104 (developed as described in Methods)

Peak calling parameters

H3K27ac: SICER or SICER2 (window size 200 bp, gap size 400 bp, E-value threshold 100)
SETD5-V5: SICER or SICER2 (window size 200 bp, gap size 600 bp, E-value threshold 100)
H3K4me1: SICER2 (window size 200bp, gap size 400 bp, E-value threshold 100)
SETD5: SICER or SICER2 (window size 200 bp, gap size 600 bp, E-value threshold 100)

Data quality

Number of peaks in each sample using above peak calling parameter:
L1_E_D0_H3K27ac_RepA: 32,231 (SICER), 33,704 (SICER2)
L1_E_D2_H3K27ac_RepA: 29,777 (SICER), 31,292 (SICER2)
L1_SETD5_D0_H3K27ac_RepA: 37,452 (SICER), 39,949 (SICER2)
L1_SETD5_D2_H3K27ac_RepA: 31,146 (SICER), 39,068 (SICER2)
L1_E_D0_H3K27ac_RepB: 35,460
L1_E_D2_H3K27ac_RepB: 33,131
L1_SETD5_D0_H3K27ac_RepB: 36,604
L1_SETD5_D2_H3K27ac_RepB: 39,451
L1_0h_SETD5-V5_RepA: 15,245 (SICER), 16,054 (SICER2)
L1_6h_SETD5-V5_RepA: 32,043 (SICER), 35,573 (SICER2)
L1_0h_SETD5-V5_RepB: 6,666
L1_6h_SETD5-V5_RepB: 27,220
L1_D0_H3K4me1: 50,276
L1_D2_H3K4me1: 49,926
L1_0h_SETD5: 1,930 (SICER), 2,132 (SICER2)
L1_6h_SETD5: 3,179 (SICER), 3,293 (SICER2)

Software

MACS1.4.2; MACS2.2.7.1; SICER1.1; SICER2-1.0.2; DAVID6.7; Homer4.10.4; CASSAVA1.8.2, Bowtie2.3.4.3, Trimmomatic0.39; Samtools1.9; Bedtools2.27.1; DEseq2.1.30.1

Flow Cytometry

Plots

Confirm that:

- [x] The axis labels state the marker and fluorochrome used (e.g. CD4-FITC).
- [x] The axis scales are clearly visible. Include numbers along axes only for bottom left plot of group (a 'group' is an analysis of identical markers).
- [x] All plots are contour plots with outliers or pseudocolor plots.
- [x] A numerical value for number of cells or percentage (with statistics) is provided.

Methodology

Sample preparation

After MDI induction, the cells were treated with 0.05% trypsin for 5 minutes at 37 degrees Celsius. Harvested cells were washed by PBS and fixed with 70% ethanol at 4 degrees Celsius overnight. Next day the cells were washed by PBS three times and stained by Cell Cycle Assay Solution Blue (Dojindo).

Instrument

LSRFortessa (BD Bioscience) was used for flow cytometry data collection.

Software

Data were analyzed using FlowJo v10 (BD Bioscience).

Cell population abundance

20,000 cells were analyzed for each condition.

Gating strategy

Cells were gated subsequently by FSC-A/SSC-A, FSC-H/FSC-W, and SSC-H/SSC-W. Then, DNA content of the gated cells were

measured. Detailed gating strategy is provided in Supplementary Figure 3a.

☒ Tick this box to confirm that a figure exemplifying the gating strategy is provided in the Supplementary Information.
